# School-based high-intensity interval exercise program in children with overweight induce a greater improvements in body composition and physical fitness than moderate-intensity continuous exercise

**DOI:** 10.1186/s12889-023-17149-7

**Published:** 2023-11-09

**Authors:** Chongwen Zuo, Xiaoyan Ma, Yuan Yang, Yupeng Cui, Chaoqun Ye

**Affiliations:** 1 Air Force Medical Center of Chinese PLA, Beijing, 100142 China; 2 School of Kinesiology and Health, Capital University of Physical Education and Sports, Beijing, 100191 China; 3 Key Laboratory of Smart Grid of Ministry of Education, Tianjin University, Tianjin, 300072 China; 4 Beihang University, Beijing, 100191 China

**Keywords:** High-intensity interver exercise, Moderate-intensity continuous exercise, Overweight, Physical fitness, Body composition, VO2max

## Abstract

**Background:**

High-intensity interval running exercise (HIIE) is emerging as a time-efficient exercise modality for improving body composition and fitness in comparison with moderate-intensity continuous aerobic exercise (MICE); however, existing evidence is still unclear in children with overweight and thus we compared the effects of HIIE and MICE on body composition, muscular, and cardiorespiratory fitness in children with overweight.

**Methods:**

In this randomized study, 40 male children with overweight aged 7–10 years were divided into an 8-week exercise regime: (1) HIIE group [n = 20; 2 sets of 15 × 20s at 85–95% maximal aerobic speed (MAS) separated by 15 × 20s recovery at 50% MAS, 3 days per week] and (2) MICE group [n = 20; 30 min at 60–70% MAS, 3 days per week]. Body composition, muscular and cardiorespiratory fitness were assessed before and after the 8-week intervention at similar times and conditions of the day.

**Results:**

Following the 8-week HIIE protocol, weight, BMI, and fat mass decreased significantly (weight: − 1.4% vs. 0.2%, *p* < 0.05; BMI: − 3.1% vs. − 0.7%, *p* < 0.05; fat mass: − 7.7% vs. − 1.6%, *p* < 0.01) as compared with MICE; while the VO2peak and MAS increased significantly in both groups, the increase in HIIE group was significantly greater than that of MICE group (VO2peak: 10.3% vs. 3.5%, *p* < 0.01; MAS:7.7% vs. 4.5%, *p* < 0.05). Although significant improvements in muscular fitness were observed in HIIE and MICE groups [counter movement jump (CMJ): 7.8% vs. 5.4%; sprinting ability: − 3.7% vs. − 1.7%], no significant differences were seen between them (*p* > 0.05).

**Conclusion:**

Our findings suggested that school-based HIIE intervention was highly in improving body composition and cardiorespiratory fitness of children with overweight than the MICE regime; however, MICE still provided improvements over time that were just not to the same magnitude of HIIE.

## Background

Obesity is a serious chronic disease that has become a global public health burden [1], and the prevalence of overweight and obese rate is still growing worldwide [2]. The world health organization (WHO) reported that due to a decline in physical activity among children and adolescents, a higher prevalence of overweight or obesity was observed in younger ages [1], who are likely to induce obesity into adulthood and more likely to develop several metabolic complications [3]. Therefore, lifestyle modification(e.g., exercise interventions) is extremely important and recommended approach to reduce adiposity during childhood and adolescence [4]. The WHO recommended moderate-intensity continuous exercise (MICE) or 60 min of moderate to high intensity physical activity per day for global children and adolescents (5–17 years) to improve body composition and physical fitness [4, 5]. However, the training duration of MICE could be as long as possible to achieve ideal results, but its longer duration is one of the main reasons why overweight or obese adolescents have difficulty in adhering due to monotony [6].

Although MICE has been proven as the most common exercise to improve body composition [7], cardio-metabolic risk factors and physical fitness [8], high-intensity interval exercise (HIIE) has become the most popular in the health and fitness industry at a global level, according to the newest report published by the American College of Sports Medicine [9]. Also, some studies under laboratory conditions [10, 11] or school-based setting [12, 13] showed that HIIE is more time-efficient than MICE in improving body composition and other health parameters of children with overweight or obesity. HIIE interventions consist of several high-intensity exercise bouts interspersed with low-intensity active or passive recovery periods between the exercise sets. Furthermore, the exercise duration and recovery periods vary from 10s to 5 min, and most protocols were conducted at an intensity > 85% of maximal heart rate (HRmax). Low-intensity recovery was adopted at 50% HRmax between HIIE bouts [10]. The work-to-rest ratio of interval exercises varied among different studies. However, HIIE is characterized by time-saving efficiency than MICE, while producing a comparable beneficial adaptation. For example, Corte de Araujo et al. [10]reported that HIIE twice a week, (e.g., 3–6 sets of 60s sprint at 100% of the peak velocity with 3 min active recovery) and MICE (e.g., 30 min efforts at 80% peak HR) for 12-week programs were equally effective in lowering the insulinemia and HOMA-index, and reducing BMI and percentages of fat mass in children with obesity. However, there is also several studies which have investigated the effects of HIIE on anthropometric indices of health [14–16]and fitness markers [17–19] in young children with overweight or obesity, but most results suggest that HIIE is equally or and in some cases more effective in improving the physical fitness than MICE [19]. Since the findings mentioned above for HIIE protocol are from children and adolescent studies with comparisons between HIIE and low-or moderate-intensity exercises, it is still unclear whether this effect can be duplicated in school setting.

Significantly, the entertainment and enjoyment of children during and after HIIE are very important for long-term exercise adherence [20]. A previous study reported that exercise intensity was negatively related to exercise adherence in children with overweight [21], but lower ratings of perceived exertion were observed when performing interval exercises in comparison to MICE [6], that further improved the exercise adherence of children. Thus, interval exercises might be more suitable for children because their activity patterns are naturally intermittent [22]. Moreover, Meng et al. [13]found a greater increase in VO2 max of obese adolescent boys with HIIE. Other studies indicated that both MICE and HIIE induced a similar increase in VO2 max [10] and a change in body composition [13] in children with obesity. However, no study has compared the effects of HIIE and MICE on physical fitness in children with overweight to directly measure the efficacy of each exercise pattern to date. School was the most ideal environment to carry out physical activity programs targeted at students’ learning and growing, allow children to keep exercise habit through physical activity interventions performed in schools, and also allowing them to take the initiative to participate in physical activity out of school (e.g., community sprots). Some of the school-based HIIE studies of children with obesity showed that HIIE programs could be performed together with physical education class or in specific period during school days [23]. Real-school environment effectiveness studies are needed that trial low-cost, convenient HIIE protocols over the long-term. Given that HIIE has been acknowledged as a time-efficient exercise and required a small physical space in non-laboratory setting and easier to perform in school, where is an appealing place.

Therefore, this study aimed to investigate the effect of two 8-week of school-based exercise training modalities (HIIE and MICE) on (a) body composition, (b) muscular fitness, and (c) cardiorespiratory fitness (CRF) assessments in children with overweight. We hypothesized that HIIE would produce more effects than MICE for improving body composition, and there would be a corresponding increase in muscular fitness and CRF because half of total training time is spent working at greater than 80% HRmax during HIIE, which could produce a considerable beneficial adaptation.

## Methods

### Study design

A randomized parallel controlled trial was adopted, with an 8-week exercise intervention period. After baseline testing, all children were randomly assigned to HIIE(n = 20) and MICE(n = 20) groups. Body composition (weight, BMI, percentages of body fat, fat and lean body mass) and several physical fitness components were assessed by investigator at baseline and after 8 weeks of exercise training at the same time with similar conditions, including muscular fitness [countermovement jump (CMJ) and 50-m sprint), and cardiovascular fitness [peak oxygen uptake (VO2peak), maximal aerobic speed, blood pressure, and HRmax]. The body composition was primary outcome, while cardiovascular fitness and muscular fitness were secondary outcome.

**Fig. 1:**
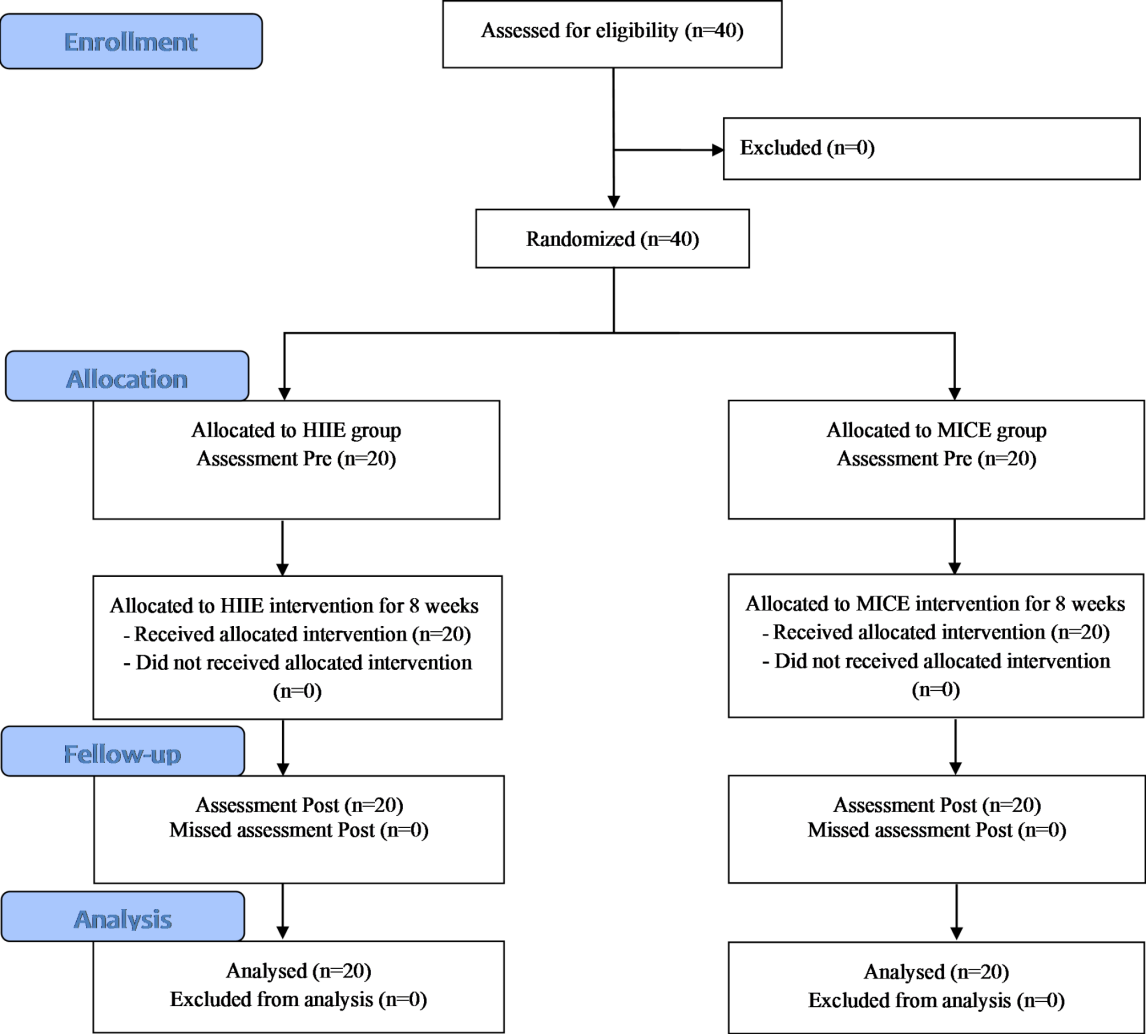
Flow chart depicting participant recruitment, randomized grouping and final analysis

Before the training, the children were assessed in two parts separated by 1 day on weekends. The first part involved body composition and graded cardiovascular fitness evaluations, and were carried out in a warm cardiovascular fitness test laboratory located in the Capital University of Physical Education and Sports. Second, the measurement of muscular fitness, Yo-Yo intermittent endurance test and training intervention were completed in the track and field of children’ school. The post-test was performed two days after the last session, and all children should complete each assessment according to the previous sequence in their free time on weekends. All children have completed at least 80% of the minimum requirements of the training sessions for inclusion in the data processing. The training protocol was approved by the Ethics Committee of the Capital University of Physical Education and Sports (Approval NO. CUPES-2018-10-15-01) in accordance with the Declaration of Helsinki. The current study design, conduct and data analysis of this trial follow the recommendations of the CONSORT statement (see Fig. 1) [24]. All children and their parents were informed about the study’s purpose, and written consent was obtained before the study commenced.

### Participants

Forty male children with overweight aged 7–10 years (including 20 classes, 2 children per class) were recruited from a local primary school in Haidian District, Beijing, China. Children were randomly assigned to HIIE (n = 20; 137.9 ± 7.9 cm; 41.5 ± 7.6 kg; 21.6 ± 1.8 kg/m^2^) and MICE (n = 20; 133.4 ± 6.8 cm; 38.4 ± 4.2 kg; 21.6 ± 1.4 kg/m^2^) groups. In this study, Randomization was carried out after collecting all baseline assessments by investigator. A simple randomization method was done to create random numbers by a blinded Excel software, which was performed by the statistics researcher. Owing to the feature of the intervention, all children and trainer might not be blinded to group assignment after randomization. Furthermore, both groups performed regular physical education classes (three sessions per week) plus the associated training interventions. The eligibility criteria were: (1) participant’s age between 7 and 10 years; (2) BMI classification for children with overweight or obesity were based on a previous study [25]; (3) absence of any orthopedic injuries and cardiovascular diseases, and (4) those with no extra regular exercise training in the last six months. Children were instructed to maintain their normal daily routines and not to participate in any extra sports training throughout the 8-week control period except their physical education classes.

### Anthropometry and body composition assessment

Before and after the 8-week exercise intervention, height and weight were measured with a portable stadiometer and an electronic scale. The total body scans were performed indoors by using the bioelectrical impedance method (JAWON; GAIA KIKO, Korea) under a controlled temperature of 24–28 °C, including the percentages of body fat as well as fat mass and lean body mass, with children wearing light clothes and no shoes. Anthropometric and body composition measurements of all children who refrained from drinking and eating for at least 12 h were simultaneously assessed before and after the intervention. This detection method is considered effective in measuring body composition changes accurately [26].

### Muscular fitness assessment

#### Lower-limb power

The Quattro Jump System (Kistler 9290AD, Switzerland) was used to assess the lower-limb power. Maximal vertical jump height was recorded to the nearest 0.1 cm after performed a counter movement jump (CMJ) without swinging the arm from the force plate. Specifically, children stood straight on the portable force plate with their hands on both sides of the hip joint as the initial position, and, after hearing the “start” cue from the instructor, they squatted down to a 90°knee angle position and jumped straight as high as possible, with a rapid, preparatory downward eccentric action while keeping hands on the hip joint. As suggested by a previous study [27], each participant performed three jumps separated individually by 1 min recovery, and the best test was used for the statistical analysis. The CMJ test is a valid and reliable field test for assessment of lower-limb power [28].

### Sprinting ability

A 50-m sprint test was performed to assess their sprinting ability in which each participant undertook a 50-m sprint and passed through an automatic photocell timer (Brower Timing System, United States). When the children heard a sound signal, they began to sprint at the fastest speed, and the timer system was activated simultaneously. Two sets of photocells were fixed at the starting position and the 50-m gates. The timing results from the finishing line were recorded as the final test result. Each participant performed two sprinting tests with appropriate passive recovery between each test, and the best test was selected as the result of the statistical analysis.

### Cardiorespiratory fitness

#### Maximal graded cardiorespiratory test

Children performed a graded maximal exercise test to determine VO2peak and HRmax on a treadmill with a respiratory gas analysis system (Cortex-model Metalyzer III B Leipzig, Germany). Calibration procedures were strictly carried out according to the previous documentation [29]. Children performed a warm-up period of 5 min at 4 km/h and then slowed down the treadmill speed to stop (0 km/h) and recover for 1 min. During the formal maximal exercise test, the initial treadmill speed was 6 km/h, and the treadmill inclination was increased by 2% per min to a maximum gradient of 12–16%. Following this, the treadmill speed was intermittently increased by 0.5 km/h per min until volitional exhaustion. The VO2peak was estimated as the highest 30s average value attained before the exhaustion while the heart rate was monitored beat-to-beat using a heart rate monitor (Polar team Oh1, Polar, Kempele, Finland) to measure the HRmax.

### Yo-Yo intermittent endurance test

The Yo-Yo intermittent endurance test was carried out to measure children’ maximal aerobic speed (MAS). All children were instructed to shuttle run between two lines separated by 20 m while gradually increasing speeds controlled by audio signals emitted from a CD player, with 10s active recovery between each 20 m shuttle run [30]. According to a previous study [31], the 20 m shuttle run is a good predictor of MAS in youth. However, the test was stopped if the participant could not complete the 20 m run in the allocated time on two consecutive attempts. The final speed after completing the 20 m run was considered as the MAS (km/h).

### Blood pressure

According to the standard guidelines [32], children took an approximately 5 min rest, and a minimum of two readings of systolic (SBP) and diastolic (DBP) blood pressures were measured in a seated position via an electronic sphygmomanometer (Omron BP652, Omron Healthcare Inc., Vernon Hills, IL, USA); the mean value of two readings was used for further analysis.

### Exercise training protocols

The children in both exercise groups (MICE and HIIE) were trained three separate days per week (e.g., Monday, Wednesday, and Friday) for 8 consecutive weeks. All training sessions were performed after school on an outdoor athletic field and were supervised by a trainer or assistant trainer. Training sessions consisted of a 5 min warm-up and cool-down at approximately 50%MAS. The warm-up included 3 min of moderate-intensity jogging and 2 min stretching.

### Moderate-intensity continuous exercise

For the MICE group, children performed a 30 min continuous endurance run on the round track at approximately 60% MAS (about 55–60% HRmax). The training progression was induced by increasing the training intensity by 10% of MAS every four weeks and further increasing it to 70% for weeks 5–8. For the HIIE group, children ran at 85% MAS during the first four weeks; then the training intensity was increased to 95% MAS for weeks 5–8.

### High-intensity interval exercise

**Fig. 2:**
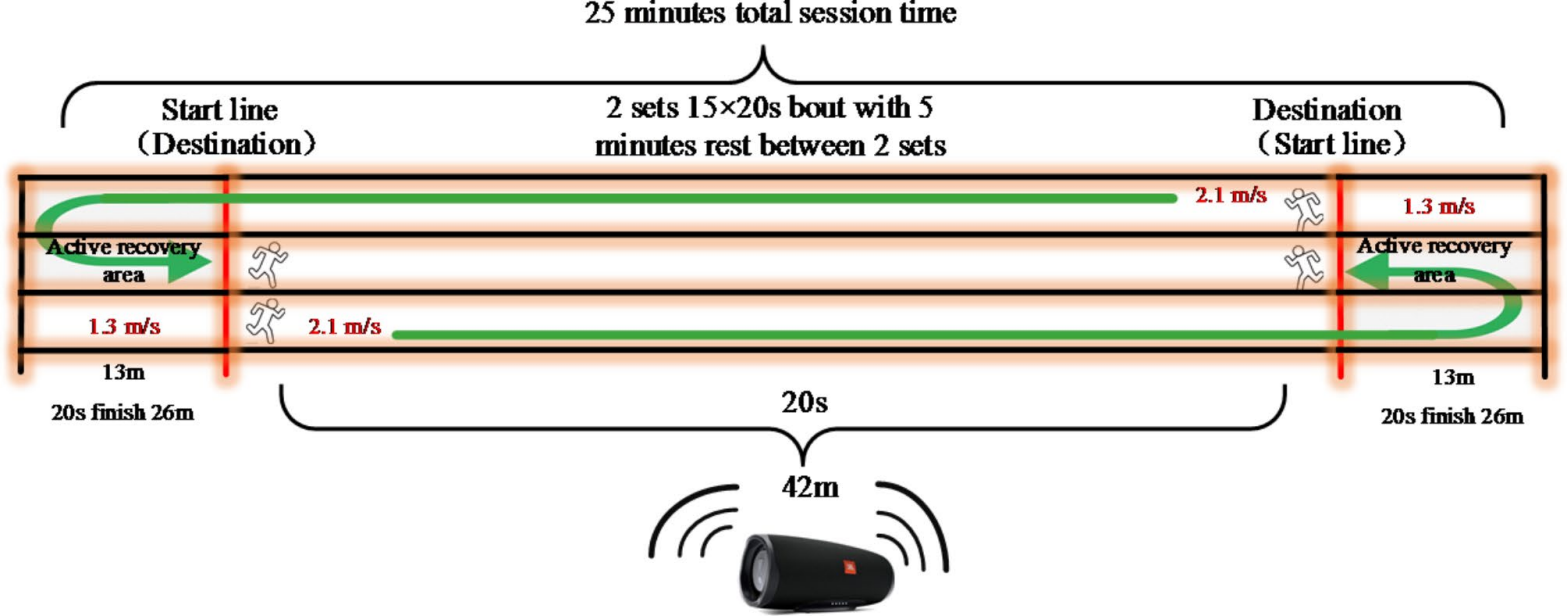
Illustration of a HIIE session including specific information about intensity, distance, sets, bouts, training time and recovery time

Children in the HIIE group were arranged in different lanes of the athletic field according to their MAS and performed 2 sets of 15 × 20s bouts of a high-intensity run (85% MAS, about 80–85% HRmax) separated by 15 × 20s active recovery bouts at low-intensity (50% MAS, about 45–50% HRmax), with 5 min rest between the 2 sets while the total duration time was 25 min. All children strictly kept the required running speed on a straight with marks track by listening to signals emitted from pre-recorded audio throughout the training session. For example, children in HIIE group may be calculated an average MAS 9.0 km/h (2.5 m/s, at 100% of MAS), they had to run 42 m in 20s (2.1 m/s, at 85% of MAS), which was followed by an active recovery to run about 26 m in 20s (1.3 m/s, at 50% of MAS) (Fig. 2). When children completed this 20s:20s bout, they turned around and ran back to repeat the remaining 14 bouts in the opposite way with the same intensity. For the MICE group, all children were encouraged to maintain the required running speed and HR by checking speed and HR data in the team watch wore on the right wrist of each participant. All training sessions in HIIE and MICE groups were controlled by a trainer and assistant trainer to ensure that each participant performed the session successfully. After four weeks of training, all children were informed to perform Yo-Yo intermittent endurance test again for speed adjustment of the training protocol. All training sessions were performed in the school setting, after class activity time, with a total of 24 sessions lasting 35–40 min per session. Both groups only took part in their normal PE classes at the school and avoided participating in additional exercise training.

### Dietary intake assessment

Daily food intake was estimated at pre-and post-intervention with validated 24-h dietary recalls (two weekdays and one weekend day). To help children accurately recall their diet, their parents recorded all consumed food items and beverages. Energy intake was analyzed by commercial software (Boohee Info Technology, Shanghai, China). All data were represented as kilocalories per day (kcal/day). All children were informed to maintain a normal diet and avoid overeating throughout the study.

### Sample size

The sample size was calculated using G*power 3.1 software (G*Power 3.1; Heinrich Heine, Dusseldorf, Germany). Using an effect size *f*^2^ = 0.25, with a power of 0.85 and a significance level of 0.05 [33], the minimum 19 subjects in each group were adequate to detect a significant difference between the two exercise groups, and thus 40 children recruited meet the sample size calculation in this study.

### Statistical analyses

Data analysis was performed using the SPSS Statistical Software version 25.0 (SPSS, Inc., Chicago, IL, USA). All baseline and post-intervention data were shown as mean ± standard deviation (SD). For paired data and unpaired data that passed or did not pass the Shapiro-Wilk’s W test for normality, a Wilcoxon signed rank test and a Mann-Whitney rank sun test were performed for baseline variables. To determine the effect of intervention on body composition, muscular fitness and cardiorespiratory fitness changes, a mixed model 2-way repeated measures analysis of variance (ANOVA) [2 times (pre and post) × 2 groups (HIIE and MICE)] was performed after checking for data normality (Shapiro-Wilk’s W test) and homoscedasticity (Levene’s test). When the interaction effect was significant, further analysis with Bonferroni post-hoc comparisons was applied. Percentage changes were also calculated and analyzed for further analysis. Additionally, Cohen’s *d* effect size (ES) was also further calculated using the eta-square (η^2^) proposed by Cohen [33] and was classified as small < 0.01, moderate = 0.01–0.138, and large > 0.138 for all data. Statistical significance was denoted at values of *p* < 0.05.

## Results

The number of children recruited in this study is showed in Fig. 1. All of the 40 children who took part in formal intervention phase. During the 8-week intervention period, nobody withdrawal from the study for personal reasons, and all children completed 24 sessions under encouragement of trainer. Therefore, a total of 40 children were analyzed (HIIE = 20; MICE = 20). The adherence to the training protocol was 100% in both groups. The main characteristics of all children are shown in Table 1. Children in both groups had similar daily caloric intakes (HIIE:2658 ± 217 kcal; MICE: 2830 ± 260 kcal, *p* > 0.05), although their caloric intake was slightly higher than the recommended daily calories (1800 ~ 2600 kcal for children) by American Heart Association [34]. The values of all anthropometric, body composition, muscular fitness, and cardiorespiratory fitness variables assessed before and after the 8-week intervention are shown in Tables 2 and 3.

**Table 1:** Physical characteristics of all children

Variables	HIIE (n = 20)	MICE (n = 20)	*p-*value
Age (years)	8.1 ±0.9	7.9 ±0.6	0.446
Height (cm)	137.9 ±7.9	133.4 ±6.8	0.064
Weight (kg)	41.5 ±7.6	38.4 ±4.2	0.124
BMI (kg/m^2^)	21.6 ±1.8	21.6 ±1.4	0.921
Body fat (%)	21.8 ±5.9	19.3 ±5.5	0.186
Fat mass(kg)	9.1 ±3.1	8.3 ±2.3	0.414
Lean body mass (kg)	29.9 ±3.0	28.1 ±3.6	0.092
Energy intake (kcal)	2658 ± 217	2830 ± 260	0.165

BMI, body mass index; HIIE, high-intensity interval exercise; MICE, moderate-intensity continuous exercise

**Table 2:** Body composition parameters for HIIE and MICE groups pre-and post-8-week training intervention

Variables	Group	Pre	Post	Interaction	*p*	η^2^
Weight	HIIE	41.5 ±7.6	40.9 ±7.4*	Time	0.114	0.064
	MICE	38.4 ±4.2	38.5 ±4.2	Group	0.165	0.050
				Time × Group	0.031	0.116
BMI (kg/m^2^)	HIIE	21.6 ±1.8	21.0 ±1.7**	Time	0.000	0.304
	MICE	21.6 ±1.4	21.4 ±1.1	Group	0.670	0.005
				Time × Group	0.014	0.149
Body fat (%)	HIIE	21.8 ±5.9	21.1 ±5.1*	Time	0.005	0.188
	MICE	19.3 ±5.5	18.9 ±5.2	Group	0.182	0.046
				Time × Group	0.597	0.007
Fat mass (kg)	HIIE	9.1 ±3.7	8.4 ±3.4**	Time	0.000	0.403
	MICE	8.3 ±2.3	8.1 ±2.3	Group	0.573	0.008
				Time × Group	0.001	0.242
Lean body mass (kg)	HIIE	29.9 ±3.0	30.1 ±3.2	Time	0.361	0.022
	MICE	28.1 ±3.6	28.2 ±3.5	Group	0.080	0.078
				Time × Group	0.762	0.002

BMI, body mass index; η^2^ eta square; MHR, maximal heart rate; HIIE, high-intensity interval exercise; MICE, moderate-intensity continuous exercise. * *p* < 0.05, ** *p* < 0.01 within group

**Table 3:** Muscular and cardiorespiratory fitness parameters for HIIE and MICE groups pre-and post-8-week training intervention

Variables	Group	Pre	Post	Interaction	*p*	η^2^
CMJ (cm)	HIIE	21.2 ±2.3	23.0 ±2.5*	Time	0.000	0.729
	MICE	21.5 ±2.7	22.8 ±3.0*	Group	0.954	0.000
				Time × Group	0.079	0.079
Sprint ability (s)	HIIE	10.8 ±0.9	10.4 ±0.7*	Time	0.000	0.281
	MICE	10.6 ±1.3	10.4 ±1.2*	Group	0.842	0.001
				Time × Group	0.201	0.043
VO2peak (mL/kg/min)	HIIE	28.9 ±2.7	32.3 ±3.2*#	Time	0.000	0.736
	MICE	29.1 ±2.6	30.2 ±3.1*	Group	0.303	0.028
				Time × Group	0.000	0.409
HRmax (bpm)	HIIE	202.9 ±5.5	197.3 ±4.4*	Time	0.000	0.604
	MICE	203.1 ±5.8	199.0 ±4.2*	Group	0.545	0.010
				Time × Group	0.229	0.038
MAS (km/h)	HIIE	9.0 ±0.9	9.8 ±0.7**##	Time	0.000	0.698
	MICE	8.6 ± 0.8	9.0 ±0.8**	Group	0.024	0.543
				Time × Group	0.010	0.163
SBP (mmHg)	HIIE	127.1 ±4.6	121.6 ±3.7*	Time	0.000	0.624
	MICE	126.3 ±4.5	122.5 ±3.6*	Group	0.983	0.000
				Time × Group	0.170	0.049
DBP (mmHg)	HIIE	80.8 ±8.2	78.8 ±7.8*	Time	0.000	0.331
	MICE	77.3 ±6.8	74.1 ±4.8*	Group	0.060	0.090
				Time × Group	0.348	0.023

CMJ, counter movement jump; DBP, diastolic blood pressure; η2, eta square; HRmax, maximal heart rate; MAS, maximal aerobic speed; HIIE, high-intensity interval exercise; MICE, moderate-intensity continuous exercise; SBP, systolic blood pressure. * *p* < 0.05, ** *p* < 0.01, within group; # *p* < 0.05, ## *p* < 0.01, between the groups

### Anthropometry and body composition

**Fig. 3:**
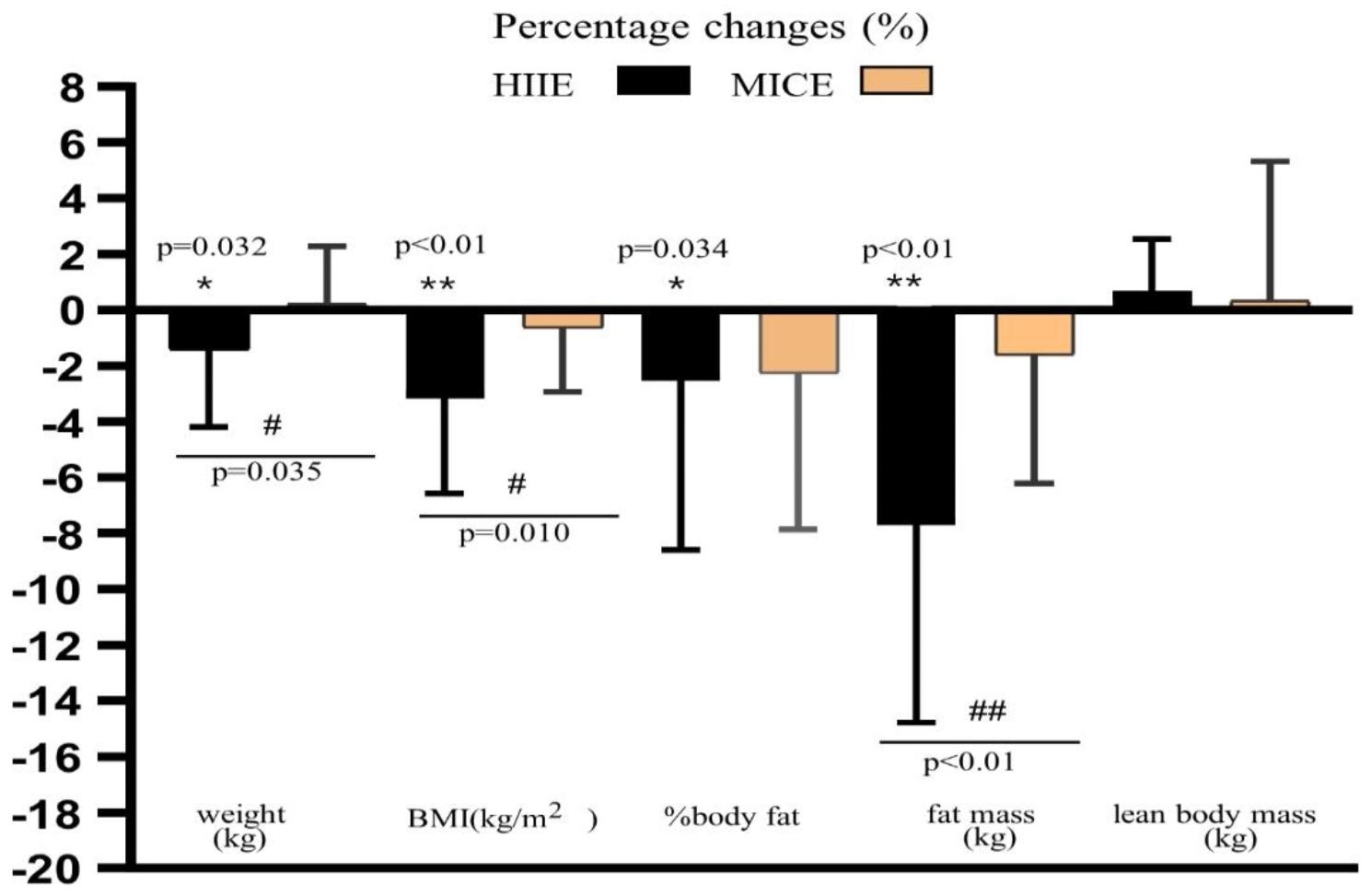
Exercise-induced changes in weight, BMI, % body fat, fat mass and lean body mass in both groups after the intervention. Note: BMI, body mass index; HIIE, high-intensity interval exercise; MICE, moderate-intensity continuous exercise; * *p* < 0.05, ** *p* < 0.01, within group; # *p* < 0.01, between the groups

Table 2 shows the results of anthropometric and body composition parameters in the groups. There was no difference between HIIE and MICE on the baseline for weight, BMI, percentage of body fat, fat mass, and lean body mass; however, a significant effect of time × group interactions on weight (*p* < 0.05, η^2^ = 0.116), BMI (*p* < 0.05, η^2^ = 0.149), and fat mass (*p* < 0.01, η^2^ = 0.242) was observed. A significant reduction in weight, BMI, percentage of body fat and fat mass from pre to post match was observed for HIIE group (*p* < 0.05), and no significant change for MICE group was verified. Additionally, in a group comparison, the percentage changes of weight, BMI, and fat mass in the HIIE group showed a significant difference (*p* < 0.05) from the MICE group (Fig. 3).

### Muscular fitness

The mean values for lower limb power and sprinting ability tests are mentioned in Table 3. Before intervention, no significant difference in CMJ and sprint tests were observed between HIIE and MICE groups. After 8-week intervention, both groups experienced a significant increase compared with pre-intervention (*p* < 0.05), with no significant difference between the groups. In addition, no significant effect of time × group interactions were observed for CMJ (*p* = 0.079, η^2^ = 0.079) and sprint (*p* = 0.201, η^2^ = 0.043).

### Cardiorespiratory fitness

As shown in Table 3, there was a significant effect of time × group interactions and on the VO2peak (*p* < 0.01, η^2^ = 0.409) and MAS (*p* < 0.05, η^2^ = 0.163), respectively. A significant increase in VO2peak (*p* < 0.05) and MAS (*p* < 0.01) from pre to post match was observed in both HIIE and MICE groups. Additionally, VO2 max (*p* < 0.05) and MAS (*p* < 0.01) was higher for HIIE group compared to the MICE group, only at the post-intervention time. Moreover, we also found a significant decrease in the HRmax, SBP, and DBP of the HIIE and MICE groups (*p* < 0.05), but there were no significant differences between the groups.

## Discussion

The main purpose of the present study was to compare the effects of school-based HIIE versus MICE on physical health-related parameters in children with overweight. The main findings demonstrated that school-based HIIE intervention was more beneficial in improving the body composition, VO2peak and MAS as compared to MICE. Moreover, no significant difference was observed in the improvement of muscular fitness between HIIE and MICE interventions.

Overweight and obesity are external forms of excess fat accumulation and storage, as well as the result of excessive energy storage [35]. However, exercise programs are one of the most sustainable and effective methods to prevent fat accumulation because they increase the body’s energy metabolism level. The current study reported that only HIIE intervention for 8 weeks significantly improved body composition and reduced weight, BMI as well as fat mass in children with overweight. Although there were no significant differences in body composition at baseline and post-intervention between the two training interventions, HIIE had a more pronounced tendency to reduce weight, BMI, and fat mass (decreases ~ 1.4%, 3.1%, and 7.7%, respectively), while MICE showed no obvious effect on these parameters. Interestingly, both HIIE and MICE interventions could not enhance lean body mass. Overweight or obesity is an abnormal accumulation or excess of fat that can cause negative effects, especially visceral adipose tissue, which is strong linked to cardiovascular diseases [36]. A previous study [37] compared the effects of MICE and HIIE on obese adolescents and reported that reduced BMI and waist circumference were more significant following HIIE than MICE. Moreover, we considered that HIIE might activate the preferential oxidation of central adiposity. Some previous studies also support our findings, indicating that HIIE is superior to MICE in improving body composition and anthropometric variables [38–40]. Although a 0.3 kg difference in fat mass between groups post intervention or 0.4 difference in BMI was observed, the using of HIIE intervention did perform ideal results as we expected, and the protocol was taken up less time than MICE. Nowadays, under the great pressure of study and after-school remedial classes, if overweight students could get extra physical exercise in less time in school, it is undoubtedly right for their health. In our study, the weight reduction might be related to a decrease in fat mass, and as total intervention time was not different between the two protocols with running time lower in the HIIE group, we suggest that the decrease in the percentage of body and fat mass in HIIE group is related to the higher energy consumption during the exercise. Moreover, Hazell et al. [41]reported that HIIE could induce greater fat oxidation capacity and higher post-exercise oxygen consumption (PEOC), which is the most likely factor leading to body fat mass reduction following HIIE interventions. Islam et al. [42] also demonstrated that acute exercise training enhanced PEOC and fat utilization, which exhibited an intensity-dependent tendency with a greater impact following HIIE than MICE. Therefore, the current study data show that school-based HIIE intervention lasting 8 weeks (3 × 25 min sessions/week) could lead to a significant improvement in the body composition of children with overweight.

Muscular fitness is considered one of the main fitness components in maintaining overall health and is usually inversely associated with overweight or obesity. Contrary to our hypothesis, HIIE was not superior to MICE for improving muscular fitness. Our study is the first study to investigate the effect of school-based interventions on the CMJ and 50 m sprint ability in children with overweight, as well as compare the effects of 8-week HIIE and MICE that indicated that both interventions were equally beneficial in promoting the lower-limb power sprinting ability. In contrast to our results, a previous meta-analysis [43] reported lesser benefits of HIIE on muscle power due to a lack of training specificity in HIIE protocols that predominantly involved continued running or sprinting without focusing on vertical power exercise, but it was more likely to improve running speed. Indeed, HIIE and MICE groups showed smaller improvements in CMJ (7.8% and 5.4%) and 50 m (3.7% and 1.7%) sprint ability. One reason for the slight higher of improvement in CMJ and 50 m sprint tests in the HIIE group than the MICE in this study, could be partially correlated to the changes in body weight [30]. Our results also verified the above-mentioned theory that the increase in CMJ performance was greater than the decrease in body weight, and the improvement in lower-limb power was higher in HIIE than in the MICE group, which could be attributed to the significant improvement of body weight and composition in HIIE group.

Children with overweight and obesity possessed lower CRF than normal-weight children, which increased the risk of cardiovascular diseases [44]. However, CRF improvement after physical training can significantly reduce cardiovascular disease and premature mortality [45]. Recent evidence of the effectiveness of various exercise types [46], including HIIE has also emphasized the importance of improving several cardiometabolic health-related parameters, including body composition and CRF in individuals with overweight/obesity [47]. Our study results suggested that the CRF was significantly improved in both HIIE and MICE groups of children with overweight following an 8-week school-based running intervention. While both running protocols could induce significant effects on CRF, the magnitude of increase in VO2peak and MAS were greater following HIIE intervention (increase ~ 10.3% and 7.7%) than MICE (increase ~ 3.5% and 4.5%). Some similar studies were also consistent with our results [16, 48, 49], suggesting that school-based HIIE interventions could elicit large improvements in CRF of children with overweight as long-term HIIE has been provided aerobic adaptations for the cardiovascular and skeletal muscle system [50]. Moreover, HIIE greatly improved cellular and peripheral vascular functions [50], which further promotes the enhancement of cardiovascular function to achieve higher aerobic workloads. Additionally, a school-based study regarding the effects of HIIE and MICE on aerobic capacity reported that HIIE increases maximal oxygen consumption to a greater degree than MICE [13]. A possible mechanism might be that HIIE provides greater stimulation of cardiac output to increase gene expression involved in skeletal muscle mitochondrial biogenesis and oxidase regulation compared to MICE [51]. However, we suggest that central adaptations to exercise training are not different between HIIE and MICE interventions, and greater increases in mitochondrial volume and function following an 8-week HIIE intervention compared to MICE might have induced increased oxygen utilization at a specific cardiac output and therefore, higher maximal oxygen consumption was produced [52].

To our knowledge, the strength of this study was the feasibility of HIIE protocol in school setting, which was not limited by the equipment and venue. The ability to conduct 20-m shuttle run and maximal aerobic speed (MAS) to assess training intensity also contributes to optimize the running programs design and obtain more improvement in body composition and CRF parameters. While there are some limitations should be acknowledged. Firstly, only boys were recruited in this study, so the subsequent studies should focus on both sexes and the more obesity groups to determine the specific effects of HIIE and MICE. Secondly, we did not monitor the daily physical activity level of each participant with a questionnaire or accelerometer, which may improve the quality of such studies if these measurements are later implemented in the future.

## Conclusion

In summary, the present study showed that an 8-week school-based HIIE program was highly in improving body composition and cardiorespiratory fitness when compared with MICE, and have a similar effect on enhancing muscular fitness of children with overweight; however, MICE still provided improvements over time that were just not to the same magnitude of HIIE. Future research is that school should consider implementing HIIE program performed in large sample sizes that have been supported while ensuring safety to promote fitness improvements in children with overweight and obesity. Moreover, school could perform HIIE program during recess to overcome the shortcoming of insufficient time and improve the chance of long-term adherence for inactive individuals.

## Data Availability

All data generated and analyzed during this study are included in this published article. The raw data supporting the conclusions of this article will be made available by the authors, without undue reservation, to any qualified researcher. First author should be contacted if someone wants to request the data from this study.

## Abbreviations

BMI: Body mass index;
CMJ: Counter movement jump;
CRF: Cardiorespiratory Fitness;
DBP: Diastolic blood pressure;
DBP: Diastolic blood pressure;
HIIE: High-intensity interval exercise
HRmax: Maximal heart rate;
MAS: Maximal aerobic speed;
MICE: Moderate-intensity continuous exercise;
VO2peak: Peak oxygen uptake;
